# Humic Acid Improves Zn Fertilization in Oxisols Successively Cultivated with Maize–Brachiaria

**DOI:** 10.3390/molecules26154588

**Published:** 2021-07-29

**Authors:** Everton Geraldo de Morais, Carlos Alberto Silva, Keiji Jindo

**Affiliations:** 1 Soil Science Department, Federal University of Lavras, Lavras 37200-000, Brazil; evertonmoraislp@gmail.com; 2 Agrosystems Research, Wageningen University & Research, P.O. Box 16, 6700 AA Wageningen, The Netherlands

**Keywords:** organometallic complexes, tropical soils, humic fertilization, residual zinc

## Abstract

Zinc (Zn) is an essential micronutrient for plant growth, and Zn deficiency is a global issue, especially in tropical soils. This study aimed to investigate the effects of humic acid (HA) and the Zn addition (Zn sulfate + HA) on the growth of maize and brachiaria in two contrasting Oxisols. The potential complexation of Zn sulfate by HA was evaluated by Fourier-transform infrared (FTIR) spectroscopy analysis. Zinc content and its availability in solution and the shoot and root biomass of maize and brachiaria were determined. FTIR spectroscopy revealed the complexation of Zn sulfate by HA through its S and C functional groups. In both Oxisols, solution Zn increased due to the combined use of Zn and HA. In a soil type-dependent manner, maize biomass and Zn in its shoots were affected only by the exclusive use of Zn fertilization. In the Yellow Oxisol, brachiaria growth and Zn accumulated in its shoot were positively affected by the combined use of Zn fertilization with HA. In the Oxisol with lower organic matter content, HA can assure adequate supplying of residual Zn, while increasing growth of brachiaria cultivated in sequence to maize.

## 1. Introduction

Zinc (Zn) plays a role in plant metabolism, acting as catalytic and structural components of proteins, tryptophan, carbohydrate metabolism, and indoleacetic acid synthesis, while maintaining cell membrane integrity and increasing lipid peroxidation [1]. Regarding tolerance to Zn, maize (*Zea mays*) is highly sensitive, while some grass plants such as brachiaria are less affected by low levels of Zn in the growth media [2]. The maize–brachiaria cultivation sequence is adopted in many Brazilian cropping areas [3]. The efficient use and adequate Zn fertilization are key issues to increase food production in Brazilian soils [2]. The Zn deficiency in soils is a global concern [2,4], mainly in tropical soils such as the Brazilian soils where parent material and intense weathering are key processes decreasing Zn availability in cerrado soils [2,4,5]. In addition, the acidic conditions, low pH, and reduced P availability in the Brazilian soils require other agronomic practices to improve crop production; liming and phosphate fertilization are frequently used to correct acidity and increase soil available P levels though these practices aggravate Zn deficiency in poor Zn fertilized cerrado soils [1,4].

In soil, Zn availability is controlled by the intensity of adsorption and precipitation reactions [4]. Zn sorption increases as the soil pH are higher due to the rise of negative charges on soil colloid surfaces and the increased presence of adsorbed and precipitated Zn forms in soil [4]. Organic matter (OM) content controls the magnitude of Zn sorption in soil [4]. OM is composed of different fractions, including humic (HA) and fulvic acids, which are OM pools characterized by low isoelectric points; thus, even at low soil pH levels, negative charges prevail on the surface of organic colloids [6]. The increase of OM and soil pH augments Zn adsorption due to the higher amount of organic ligands and the increase of density of charges in these organic ligands, besides the increased stability of organic complexes with HA, as the pH is increased [4,7]. An increase in clay content is a key factor to increase Zn adsorption in soils. However, the density of negative charges in tropical clay minerals and Al and Fe oxides is lower than those in organic colloids [1,2,4]. Moreover, Zn availability to plants is controlled by soil type, minerals associated with clay and Fe and Al oxides, soil parent material, total Zn content, soil pH, concentrations of organic matter, Ca, calcite, bicarbonate, and phosphate found in the soil, solution or in labile forms prone to be solubilized and to react with zinc with subsequent formation of precipitates and high-stability organometallic complexes [1,2,4,7].

In Brazilian cropland areas, the increase of OM and humic substances stored in the soil is not a simple task, even in no-tillage and conservative cropland fields. This is why the soil amendment with exogenous OM such as humic acid should be tested to reduce specific Zn adsorption into soil mineral colloids while improving Zn uptake by crops. Humic acid, a pool of humic substances (HS), is prone to form complexes with Zn. However, the stability and solubility of complexes formed depend on the Zn–HA stoichiometry, plant type, HA rate added to soil, growing medium properties, and soil type [7,8,9,10,11]. Therefore, depending on the combination of the factors mentioned above and soil management practices adopted, Zn uptake by crops may be improved or diminished [8,9,11]. In combination with Zn, HA addition to soil improves nutrient uptake and plant growth through direct and indirect effects of HS [9,11]. Direct effects occur when HA is in contact with the plant cell membrane and tissues, stimulating several plant chemical, physiological, and biochemical processes [11,12,13,14]. The action of HS on plant tissues includes the stimulation of root proliferation, enhancement of H^+^-ATPase activity in the cell membrane, and changes in the magnitude of nutrient uptake, assimilation, and use efficiency [8,9,11,12,13,15,16].

Indirect effects of HS are related to the modifications in the growing medium in response to humic fertilization [11,12]. In soil, HA may complex Zn [7], improving Zn nutrition and plant growth [17]. Complexation of Zn by organic ligands is relevant to improve Zn uptake by plants, considering that the organometallic complexes increase Zn content in soil solution and Zn diffusion from solution to cell root surface as well [4,7,10,14]. Even though the Zn concentration in solution is only a tiny fraction of soil total Zn content, Zn dissolved in the soil liquid phase is a readily available Zn pool for plants [4]. Therefore, it is expected that HA has organic radicals in its structure capable of complexing the Zn in soluble forms. Even at high soil pH conditions (pH 7.2), the HA uses efficiently increased the Zn content in solution [10]. 

When present in soil solution, HA is also capable of buffering soil acidity, keeping the pH in the optimum range required for plentiful nutrient supplying to crops, mainly of Zn, whose availability is reduced as the soil pH is increased [1,2,4]. The HA effect on soil acidity buffering is highlighted primarily in soils with low clay and organic matter contents. In less buffered soils, pH oscillation is more frequent, and, when pH is within the alkaline range, less dissolved Zn is found in soils, while low insoluble forms (ZnOH^+^ and ZnOH_2_) are formed in the whole soil [2,4]. When present in the growing medium, HA is capable of buffering soil acidity, avoiding oscillations in soil reaction [14,18], which, consequently, favours the formation of soluble Zn^2+^ free hydrated forms, as well as soluble Zn-organic complexes [4].

Humic acid effects on plants also rely on the properties of HA’s structure-organic functional groups-function [15,17]. The positive effects of humic acid on maize or brachiaria were already reported, as well as the residual effects of humic fertilization on brachiaria after successive cuttings [8,19]. In tropical soils, Zn is prone to be immobilized in the soil mineral phase, but little is known regarding the humic fertilization–Zn interaction on plant nutrition and growth. Additionally, in the large area of Brazilian integrated crop-livestock systems, maize and brachiaria are simultaneously cultivated [3], and both crops show different responses to HA application and Zn fertilization [2,4,8].

The direct and indirect effects of HA occur simultaneously [9,11], and are linked to the type and concentration of oxygen-, nitrogen-, and sulfur-containing functional groups in the HA structure [14,15,20]. Infrared spectroscopy is a suitable technique to reveal the functional groups and specific bonds formed between the metals and HA [6,7,14]. The use of infrared to evaluate the chemical complexation was proved when ZnCl_2_ was mixed with HA, considering that the OH-stretching at 3290 cm^−1^ increased when Zn was complexed by HA [7]. Other bonds are involved in Zn complexation through organic molecules, such as the FTIR peak at 1075 cm^−1^ related to the formation of organic-SO_4_^−2^–metal complexes [21], reflecting the role played by HA sulfur groups in complexing Zn [20]. When organic matrices and metal are mixed, HA acts both as a source of complexed metal to plants and bioactive molecules capable of improving plant nutrition, physiology, and growth [20]. Some functional groups found in the HA structure are positively related to HA bioactivity [14]. Besides, the peaks related to the C-H_3_ bending, C=C (aromatic-C), and aliphatic C–H stretching recorded in HA infrared spectra contribute to improving plant growth medium capacity to buffer acidity [14,18].

This work is the first attempt to study the HA-Zn interaction in contrasting Brazilian soils. HA is not supposed to play a role in plant growth and soil Zn chemistry, forms, and availability in OM-enriched soils. In less-buffered soils, HA is prone to act as a buffer for acidity, which may improve brachiara’s nutrition and growth, which is more adapted than maize to acidic soils. 

Zinc is prone to be adsorbed in Brazilian soils enriched with clay and Al and Fe Oxides. However, in sandy soils, the simultaneous reduction of Zn adsorption, and the formation of Zn–HA complexes should act positively on soil Zn availability to crops, mainly for brachiaria, which is not fertilized in most Brazilian pasture areas. The use of Zn in combination with HA is a promising strategy to decrease Zn adsorption and precipitation while increasing the formation of soluble Zn–HA complexes that are prone to nourish or to be acquired by roots. When combined with HA, Zn is supposed to prevail in soils in soluble forms, thus increasing its residual effect on brachiaria plants. In Brazil, the supply of Zn relies only on the Zn fertilization of previous crops (maize) in rotation. Thus, it is interesting to investigate how soil type influences Zn fertilization’s efficiency and the magnitude of HA action on plant and Zn supplying to maize and brachiaria. 

We hypothesized that when Zn sulfate (ZnSO_4_) (the main source of Zn for plants [4]) is mixed with HA, there are modifications in the HA structure due to the formation of Zn–HA complexes. Some organic functional groups of HA can act by complexing with Zn, playing a positive role in the efficiency of Zn fertilization for the maize–brachiaria rotation in a soil type-dependent manner. The aims of the study were: (1) To demonstrate the formation of Zn–HA complexes through the use of FTIR spectroscopy analysis; (2) To study the capacity of HA in increasing the effects of Zn fertilization on the nutrition and growth of brachiaria following maize cultivation; (3) To evaluate the effects of HA on the Zn availability in soil and solution, and soil residual Zn to brachiaria plants; (4) to verify if the combination of Zn–HA is effective in improving plant growth in the two constrasting Oxisols.

## 2. Results

### 2.1. Maize Growth

**Figure 1:**
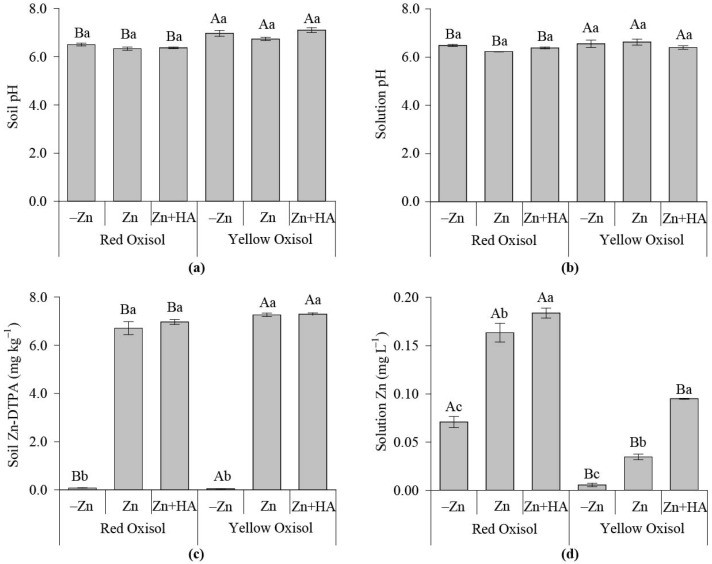
Effects of Zn fertilization and humic acid (HA) on soil pH and Zn availability in contrasting Oxisols cultivated with maize. Soil pH (**a**); soil solution pH (**b**); soil available Zn by the DTPA method (**c**); Zn in soil solution (**d**). Bars followed by the same capital letter means that the soil type properties differ regarding the Zn fertilization strategies studied according to the Tukey test (*p* < 0.05). Bars followed by the same lowercase letter mean that crop traits do not differ regarding the Zn fertilization strategies adopted, again, according to the Tukey test (*p* < 0.05), within each soil type. −Zn: no-Zn fertilization; Zn: Zn fertilization; Zn + HA: Zn fertilization combined with HA application.

The addition of HA and/or Zn did not significantly affect soil pH, as well as solution pH (Figure 1a,b). YO had higher soil and solution pH values than RO. The soil–Zn DTPA increased in response to Zn fertilization over control (no Zn added to the soil), regardless of the HA application (Figure 1c). Soil Zn-DTPA contents in the YO are significantly higher than RO. In addition, it shows that RO had a higher soluble Zn content than YO (Figure 1d). For both Oxisols, the Zn fertilization increased the solution Zn content over control. When HA was combined with Zn, the solution Zn was significantly higher than the single-use of Zn fertilization (Figure 1d).

**Figure 2:**
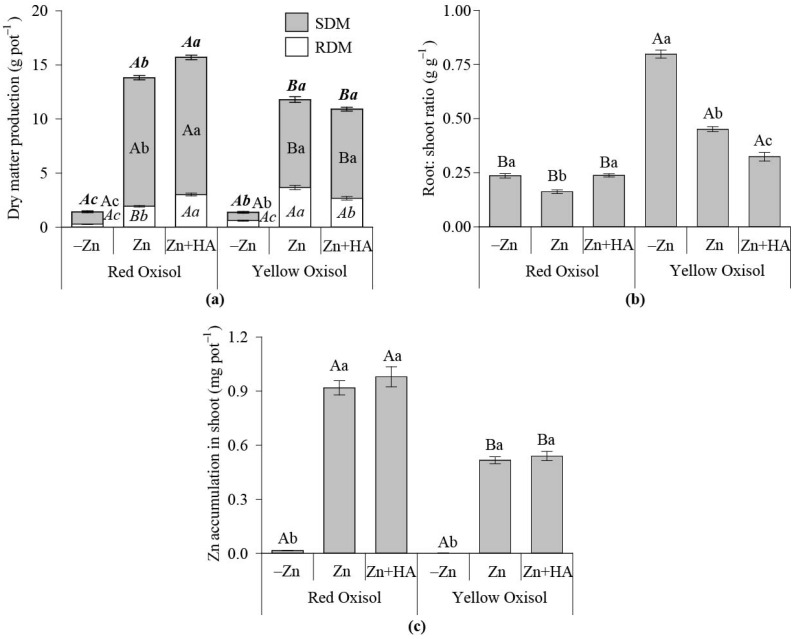
Effect of Zn fertilization and humic acid (HA) application on Zn nutrition and maize growth in contrasting Oxisols. Dry matter production (**a**); Root: shoot ratio (**b**); Zn accumulated in maize shoot (**c**). SDM: shoot dry matter; RDM: root dry matter; full bar in the biomass graph represents the total dry matter production (TDM). Bars followed by the same capital letter do not differentiate the soil types regarding the Zn fertilization strategies studied, according to the Tukey test (*p* < 0.05). Bars followed by the same lowercase letter do not differentiate the Zn fertilization strategies adopted within each soil type by the Tukey test (*p* < 0.05). The combined use of bold-italic letters compare TDM means in response to treatments; the use of italics compared RDM means in response to treatments. −Zn: no-Zn fertilization; Zn: Zn fertilization; Zn + HA: Zn fertilization combined with HA application.

In RO, the SDM, RDM, and TDM increased in response to Zn fertilization, and maize biomass production with HA combined with Zn was slightly greater in comparison to the single use of Zn (Figure 2a). SDM and TDM increased due to Zn fertilization over control, independent of the HA use in YO (Figure 2a). However, the RDM decreased in response to HA added in combination with Zn (Figure 2a). When Zn was applied to soil, maize growth is more prominent in RO over YO, mirrored by a greater SDM, RDM and TDM. YO has a significantly higher maize root: shoot ratio than RO. Root: shoot ratio reduced due to the HA application compared to the single-use of Zn fertilization in YO (Figure 2b). Zinc accumulated in maize shoot increased in response to Zn fertilization, regardless of the HA use, with higher amounts of Zn in maize shoot cultivated in YO over the plants grown in RO (Figure 2c).

### 2.2. Brachiaria Growth

**Figure 3:**
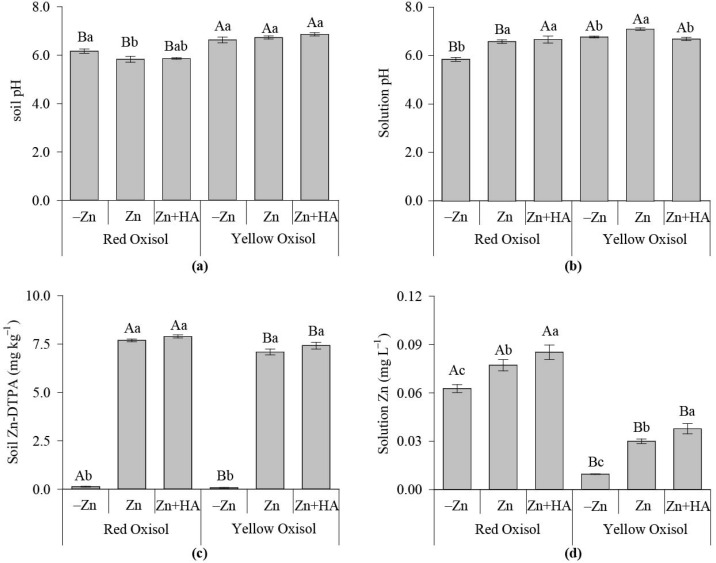
Effect of Zn fertilization and humic acid (HA) on soil pH and available DTPA Zn in contrasting Oxisols cultivated with brachiaria. Soil pH (**a**); soil solution pH (**b**); Zn available through the DTPA soil test (**c**); Zn in soil solution (**d**). Bars followed by the same capital letter mean that the soil attributes did not differ in terms of the Zn fertilization strategies adopted, according to the Tukey test (*p* < 0.05). Bars followed by the same lowercase letter mean that the soil attribute means do not differ regarding response to Zn fertilization strategies within each soil type, according to the Tukey test (*p* < 0.05). −Zn: no-Zn fertilization; Zn: Zn fertilization; Zn + HA: Zn fertilization combined with HA application.

When brachiaria was grown in sequence to maize, the soil pH was higher in YO than RO (Figure 3a). Solution pH was similar in both Oxisoils when Zn fertilization was combined with HA (Zn + HA); without HA application, YO had a greater solution pH than RO (Figure 3b). HA significantly reduced the solution pH in YO (Figure 3b). Soil Zn-DTPA content was higher in RO over YO, regardless of the use of Zn and HA. (Figure 3c). Zinc fertilization increased the soil Zn-DTPA content, independent of soil type and HA application (Figure 3c). The solution Zn was higher in RO over YO, and HA applied together with ZnSO_4_ increased the solution Zn in both Oxisols (Figure 3d).

**Figure 4:**
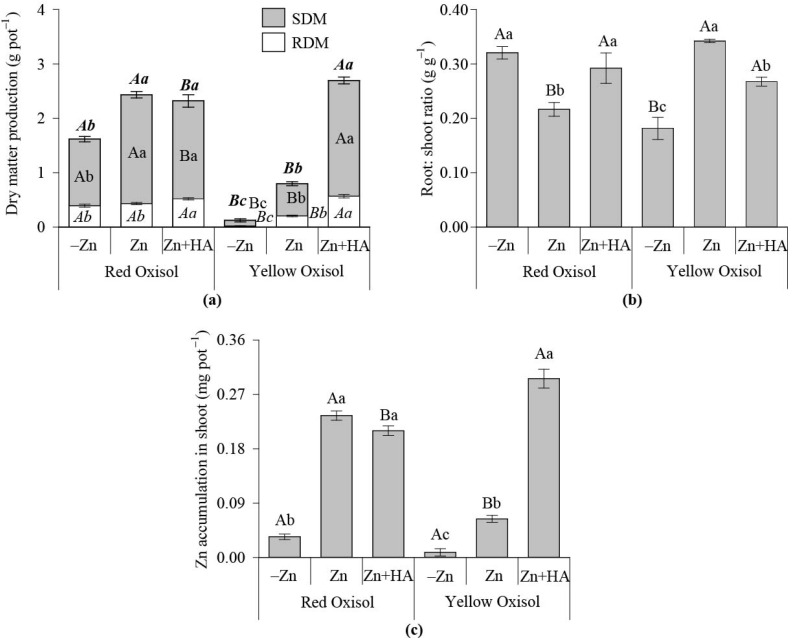
Effect of Zn fertilization and humic acid (HA) application on brachiaria growth traits and Zn nutrition of plants grown in contrasting Oxisols. Dry matter production (**a**); Root: shoot ratio (**b**); Zn accumulated in brachiaria shoot (**c**). SDM: shoot dry matter production; RDM: root dry matter production; full bar in the biomass graph represents the total dry matter production (TDM). Bars followed by the same capital letter do not differentiate the soil types according to the Zn fertilization strategies studied by the Tukey test (*p* < 0.05). Bars followed by the same lowercase letter do not differentiate the Zn fertilization system adopted to each soil type according to the Tukey test (*p* < 0.05). The combined use of bold-italic letters was used to compare TDM treatment means; the use of italics compared the values of RDM across the treatments. −Zn: no-Zn fertilization; Zn: Zn fertilization; Zn + HA: Zn fertilization combined with HA application.

The treatment of Zn+HA significantly increased SDM and TDM over control (-Zn) in RO, mainly affected by the increased RDM in response to HA combined with Zn fertilization (Figure 4a). In YO, the SDM, RDM, and TDM increased due to Zn fertilization over control (no Zn addition), and HA acts in synergy with Zn fertilization considering that HA promoted a higher SDM, RDM and TDM over the exclusive use of Zn in maize fertilization (Figure 4a). In the absence of HA, the RO over YO produced a higher maize SDM, RDM and TDM. In the presence of HA, YO over RO had a greater maize SDM and TDM (Figure 4a). In RO, HA application increased the root: shoot ratio compared to the single-use of Zn. In YO, the HA use decreased the root: shoot ratio compared to the exclusive use of Zn. Zinc accumulated in maize shoot grown in RO increased due to Zn fertilization, but it was not influenced by HA (Figure 4c). In YO, the positive effect of Zn use on Zn in maize shoot was magnified when HA was combined with Zn sulfate (Figure 4c). When compared, the plants grown in the RO over the YO had a greater Zn accumulation in the shoot in response to the single use of Zn fertilization. On the contrary, Zn fertilization in combination with HA assured the highest accumulation of Zn in maize plants grown in YO (Figure 4c).

### 2.3. Principal Component Analysis

**Figure 5:**
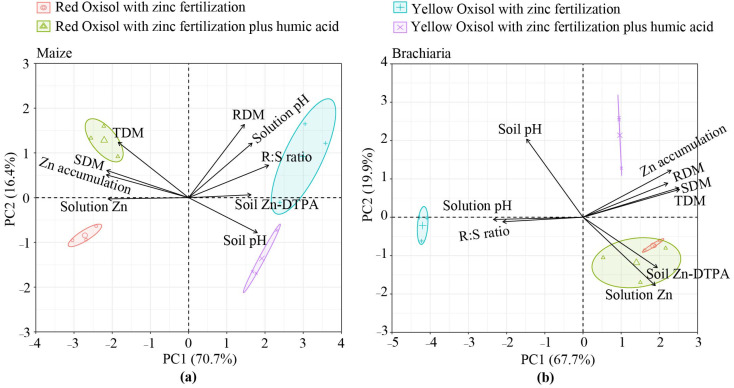
Biplot of principal component analysis (PCA) separated according to the crop grown in both Oxisols. The main clusters of plant traits and soil attributes in response to Zn with/without humic acid (HA) use are shown in PCA graphs. The maize experiment variables (**a**); brachiaria experiment variables (**b**); SDM, RDM and TDM: shoot, root and total dry matter production, respectively; R:S ratio: ratio between shoot (SDM) and root (RDM) biomasses; Zn accumulation: Zn accumulated in shoot.

By reducing dimensionality, PCA is a multivariate analysis capable of identifying potential correlations, dependencies, and features of the main clusters of a dataset in response to the Zn treatments investigated. When maize and soil attributes were analyzed, 87.1% of the covariances can be explained by the PC1 and PC2 axes, while for the brachiaria experiment, 87.6% (Figure 5a) of the variations were explained. For the maize experiment, PCA showed that the solution pH and soil pH are favoured by the absence of HA in YO. Soil pH and solution pH are negatively related to solution Zn, Zn accumulated in shoot, SDM and TDM. 

On the contrary, plant traits are favoured by the Zn fertilization combined with HA. The solution pH is positively related to RDM and root: shoot ratio for the maize trial. The significance of some correlations aforementioned was confirmed by the Pearson’s linear correlation matrix (*p* < 0.05) (Supplementary Material 1, maize experiment). Thus, soil solution pH was negatively correlated with soluble Zn, Zn in shoot, positively associated to RDM and root: shoot ratio (Supplementary Material 1); soil pH negatively correlated with soluble Zn, SDM, TDM and Zn in shoot, and positively correlated with the root: shoot ratio. Soluble Zn is directly linked to SDM, TDM, Zn shoot accumulation and negatively related to RDM and shoot: root ratio. The root: shoot ratio is negatively correlated with SDM, TDM and Zn accumulated in maize shoot.

As outcomes of PCA for the brachiaria experiment, it was verified that Zn accumulated in shoot, and the RDM, SDM and TDM were favoured by the addition of Zn+HA in the case of YO. Plant variables are negatively related to solution pH and root: shoot ratio (Figure 5b). The solution Zn and soil Zn-DTPA were related to each other and negatively associated with soil pH. To check the statistical significance and association degree of the relationships as mentioned above, the Pearson’s linear correlation matrix analysis (*p* < 0.05) was performed (Supplementary Material 2). With Pearson’s linear correlation analysis, a negative correlation between soil pH and soluble Zn and soil-Zn DTPA was confirmed. The solution pH was negatively correlated with soluble Zn, SDM, RDM, TDM, and Zn accumulated in brachiaria shoot, and positively associated with root: shoot ratio. Root: shoot ratio also was negatively correlated with SDM, TDM, and Zn in brachiaria shoot.

### 2.4. Infrared Spectroscopy

**Figure 6:**
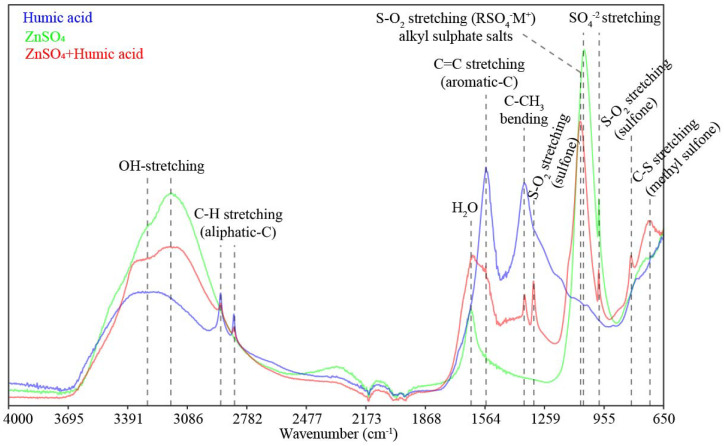
Peaks and the main features of chemical groups recorded by the Fourier-transform infrared spectroscopy analysis with attenuated total reflectance (FTIR–ATR). FTIR bands were assigned for pure samples of ZnSO_4_ (Heptahydrate zinc sulfate), humic acid and the mixture of ZnSO_4_ with humic acid.

FTIR-ATR peaks (Figure 6) in HA spectra at 3290 cm^−1^ are related to OH stretching, at 2915 and 2845 cm^−1^ to aliphatic CH groups, at 1560 cm^−1^ is assigned to C=C stretching in aromatic rings, while at 1365 cm^−1^ is related to C-CH_3_ groups bending [7,18]. Spectra of the zinc sulfate revealed peaks at 3170 cm^−1^ related to OH stretching, at 1640 cm^−1^ assigned to water, and at 1060 and 980 cm^−1^ linked to SO_4_^−^ stretching (free sulfate groups) [22,23]. When ZnSO_4_ was mixed with HA, the peaks assigned to OH stretching (3290 and 3170 cm^−1^), aliphatic CH groups (2915 and 2845 cm^−1^), water (1640 cm^−1^), aromatic-C (1560 cm^−1^), C-CH_3_ groups bending (1365 cm^−1^), and SO_4_^−^ free groups (980 cm^−1^) were the main features recorded in the HA-Zn spectra [7,22,23]. Additionally, for the ZnSO_4_+HA mixture, new peaks at 1315 and 815 cm^−1^ were related to SO_2_ stretching (sulfone) and, at 715 cm^−1^, to C-S stretching (Methyl sulfone-CH_3_-SO_2_) [21]. The infrared peak at 1060 cm^−1^ related to SO_4_^−^ stretching (free sulfate groups) from ZnSO_4_ mixed with HA shifted to 1075 cm^−1^, which is assigned for SO_2_ stretching (alkyl sulfate salts), indicating the formation of RSO_4_^−^M^+^, where R is the humic organic radical bonded to M (Zn^2+^)^+^, the complexed metal [21].

## 3. Materials and Methods

### 3.1. Maize–Brachiaria Growth Conditions

#### 3.1.1. Treatments and Growing Conditions

Effects of Zn fertilization, combined or not with HA, were tested on a sequence of maize–brachiaria grown in greenhouse conditions. The HA used in the experiment was extracted from Leonardite through a 0.05 mol L^−1^ KOH solution [24]. The main HA properties are described as follows: pH: 9.7, E4/E6 ratio: 4.84, C: 354.9 g kg^−1^, N: 5.3 g kg^−1^, Zn: 76.3 g kg^−1^ [10,17]. The Zn source was the heptahydrate zinc sulfate (ZnSO_4_·7H_2_O) *pure per analysis* reagent [p.a.]). HA and Zn were added to Oxisols (two) with clay and organic matter (OM) contrasting contents. Plants were grown in pots filled with 1.8 kg Oxisol samples. In the Red Oxisol (RO), liming was performed to increase Ca and Mg optimum levels for maize-brachiara and raise pH to 6.0. Soil samples were incubated for 30 days with CaCO_3_ and MgCO_3_ (p.a.) at the 4:1 ratio, keeping the soil moisture close to 70% of soil maximum water-holding capacity (MWHC). In the Yellow Oxisol (YO), the soil pH and Ca content were already in levels considered optimum for both maize and brachiaria plentiful growth; thus, liming was not performed, and only 15 mg kg^−1^ Mg was provided to maize at the sowing fertilization through the MgSO_4_·7H_2_O p.a salt. After soil acidity correction, soil samples were dried and passed through a 2 mm sieve and used in the lab to determine the main physical and physicochemical properties of Oxisols (Table 1).

Treatments were displaced in pots using a complete randomized design with three replicates in a 2 × 3 factorial scheme; thus, the first factor is related to soil type (RO and YO), while the second one is related to Zn fertilization strategies as follows: no-Zn fertilization and no-HA addition to Oxisols (1), only Zn fertilization (2), and Zn fertilization combined with HA application (3). Oxisols were treated with 20 mg kg^−1^ Zn and 50 mg kg^−1^ C-HA were added to soils when Zn was combined with HA. The rate of HA added to soils was based on previous studies carried out to set the optimum HA level to plentiful plant growth [9,25]. Treatments were homogeneously mixed with the whole soil mass at the beginning of maize cultivation. 

At the maize sowing, nutrients were supplied to plants at the following concentrations: 135, 300, 100, 40, 0.81, 1.33, 3.66, 0.15, and 1.55 mg kg^−1^, respectively, N, P, K, S, B, Cu, Mn, Mo, and Fe, using, respectively, the following sources: NH_4_H_2_PO_4_, K_2_SO_4_, H_3_BO_3_, CuSO_4_.5H_2_O, MnCl_2_.4H_2_O, (NH_4_)6Mo_7_O_24_.4H_2_O and FeCl_3_.6H_2_O p.a. In sequence, the soil moisture was kept at ~70% MWHC, then, five maize seeds were sowed per pot. Ten days after maize planting, thinning was performed, and two plants were left in each pot. The top-dressing fertilization was carried out at 15 and 20 days after maize planting, adding to soil 100 mg kg^−1^ N, which was provided as NH_4_NO_3_ p.a. Maize plants were grown in greenhouse conditions for 30 days.

To evaluate the Zn fertilization residual effect after the maize cultivation, ten seeds of *Brachiaria brizantha* cv. Paiaguás were planted in the same pot used to grow maize. Ten days after planting, thinning was done, and two brachiaria plants remained in each pot. The brachiaria planting fertilization was carried out by adding to soil 100 mg kg^−1^ N and 100 mg kg^−1^ K, respectively, supplied through the NH_4_NO_3_ and KCl p.a. salts. The top-dressing fertilization of brachiaria was carried out at 15, 25, and 35 days after planting, using 100 mg kg^−1^ N, which was provided to plants as NH_4_NO_3_ p.a. in each top-dressing fertilization. The brachiaria was grown for 45 days, and Zn was only furnished to maize plants; thus, brachiaria was nourished only with the residual Zn found in soils previously cultivated with maize.

#### 3.1.2. Soil and Solution Analysis

At the beginning of each cultivation, after 12 h of planting and keeping the soil moisture at ~70% MWHC, an aliquot of the soil solution (20 mL) was collected using the Suolo Acqua^®^ sampler [26]. The soil solution sampler was inserted in the middle of the pot during its filling with soil. Soil solution samples were filtered through a 0.45 µm membrane, and their pH values were determined through a Mettler Toledo bench pH meter. Zn content in soil solution (soluble Zn) was determined by inductively coupled plasma optical emission spectroscopy (ICP-OES). After 18 h of planting, it was collected 30 g of soil in each experimental unit, then the soil samples were dried and sieved (2 mm). The pH of soil samples were determined in a suspension of 1 g soil plus 2.5 mL water. Available Zn content in soil (soil Zn-DTPA) was extracted by the DTPA soil test [27], and Zn was determined in an ICP-OES machine. A sampling of solution and the whole soil was carried out for each experimental unit, thus, totaling three replicates for each treatment investigated.

#### 3.1.3. Biomass Production and Zn in Shoot

At the end of maize or brachiaria cultivation, plants were harvested, separated into shoot and root, and dried in an oven with air circulation at 60 °C until constant weight. The dried biomass was weighed, and shoot (SDM) and root (RDM) dry matter production was determined. The total dry matter was obtained by summing SDM plus RDM. The root: shoot ratio was calculated by dividing SDM by RDM. The shoot dried biomass was sieved (1-mm) and digested in a mixture of nitric and perchloric acids at a 4:1 ratio [28], and Zn content in the shoot was, in sequence, determined in an ICP-OES machine. Based on the SDM and Zn content in a shoot of each experimental unit, Zn accumulation was calculated, as follows: ZnAc (mg pot^−1^) = SDM (g pot^−1^) × ZnC (mg g^−1^), in which ZnAc is the Zn accumulated in shoot; and ZnC is the Zn content in maize or brachiaria shoots.

### 3.2. Infrared Spectroscopy

The spectra of HA and ZnSO_4_·7H_2_O samples were recorded by Fourier transform infrared spectroscopy (FTIR) with attenuated total reflectance. The same proportion of HA and ZnSO_4_·7H_2_O used to treat maize plants was dissolved in water; in sequence, the mixture was dried at 60 °C until constant weight, then, the FTIR analysis was performed. FTIR was performed in an Agilent^®^ Cary 630 spectrometer with a ZnSe crystal. The measurements were performed in the wavenumber range of 4000 to 650 cm^−1^ with a resolution of 4 cm^−1^. The FTIR spectra dataset was pre-processed using the background correction and dataset normalization procedures [29].

### 3.3. Statistical Analysis

All statistical analysis was carried out using the base, stats, agricolae, corrplot, factoextra and FactoMineR packages of the R software [30,31,32,33,34,35]. Effects of treatments on plant and soil means were compared through the Tukey test (*p* < 0.05) after the assumptions of analysis variance (ANOVA) were attended (*p* < 0.05). Before the ANOVA test, we assessed the normality distribution (Shapiro–Wilk’s Test), homogeneity (Bartlett’s Test), and independence of the observations and residuals (Durblin–Watson’s test). Principal component analysis (PCA) was performed after removing the no-Zn fertilization data from the whole dataset due to the high negative influence of the absence and severe deficiency of Zn on plant growth (biomass). In sequence, the dataset originating from the maize or brachiaria experiments was staggered separately, and PCA was performed for the dataset of each crop grown in both Oxisols. The Pearson’s linear correlation matrix (*p* < 0.05) was also carried out, aiming to validate clusters and potential relationships of soil and plant attributes as outcomes of PCA (Supplementary Material 1 and 2). Before the PCA and correlation analysis, each variable from the dataset was tested regarding its normality through the Shapiro–Wilk test, and once the normality was confirmed, the analysis was performed.

## 4. Discussion

When Zn is mixed with HA, free sulfates were kept in the complex formed (SO_4_^2−^ stretching at 980 cm^−1^) (Figure 6). Additionally, bonds related to sulfone groups (S-O_2_ stretching and C-S methyl sulfone) are formed, which is indicative of the interaction of HA-with ZnSO_4_ and synthesis of Zn–HA complexes. Sulfur groups present in the mixture of HA+ZnSO_4_ are indicative of Zn–HA complexation. This was possibly due to the presence of oxygen-, nitrogen-, and sulfur-containing functional groups in the HA structure [15,20]. Another peak indicating the Zn complexation with HA is recorded at 1075 cm^−1^, which is an FTIR band related to the formation of a new spectral signature related to RSO_4_^−^M^+^ groups, where R is the C-humic functional group and M^+^ is the complexed metal [21]. FTIR bands indicated that Zn from sulfate was complexed into the humic matrix. The peak recorded at 3290 cm^−1^ and related to OH stretching is more pronounced when ZnSO_4_ is mixed with HA, compared to the intact HA spectral signature, indicating the increase of hydration and production of Zn aqueous complexes [7]. Hydroxide ions (OH^−^) found in carboxylic and phenolic groups and aliphatic domains of HA play a key role in forming soluble metal complexes [15], resembling the spectral signature of the Zn--humic acid complex synthesized in this study.

The specific spectral signature of Zn_complexes demonstrated the potential of FTIR spectroscopy in identifying Zn sulfate bonded to HA, compared to the over bands recorded for the intact HA. The interaction of HA with Zn was relevant to show changes in functional groups of the new configuration and spectral signature of HA as an organic ligand for Zn, as well as to indicate and changes in the intensity of FTIR bands’ shift [7]. The mechanisms and processes of Zn interaction with HA included ionic or uncoordinated forms, unidentate complexes, bidentate chelates, and bidentate bridging coordination bonds [7]. The interaction of organic ligands with Zn is beneficial to Zn supplying to plants, considering that free Zn ions are prone to react with soil components through sorption and precipitation, which are the main processes regulating Zn availability in soils [4,11,16].

Zn deficiency is a global issue [2,4] and is frequently reported in tropical soils [5]. Plants have different sensitivities to Zn deficiency in the soil. While maize (*Zea mays*) has a high sensitivity, grass such as *Brachiaria brizantha* is known to tolerate Zn deficiency [2]. The natural deficiency of Zn in tropical soil limits crop production, as occurred in this study. Both maize and brachiaria growth were hampered by the limited supply of Zn in both Oxisols with contrasting OM and clay contents. Thus, regardless of the Oxisol cultivation, the maize and brachiaria biomasses are sharply reduced when the supply of Zn is restricted in both highly weathered soils (Figure 2 and Figure 4).

Zn deficiency is frequently reported in other studies conducted in low pH tropical soils with reduced P availability [5]. Zn is prone to interact with the surface of minerals found in soil colloids negatively charged, which reduces its availability to crops; liming increases the adsorption of Zn into soil colloids due to the increased density of negative charges on the surface of clay minerals and Fe and Al (hydro) oxides [2,10]. 

In this study, the reduction of soil Zn deficiency by Zn fertilization was followed by the significant effect of solution pH on solution Zn. Thus, changes in Zn availability affected SDM, TDM, and the accumulation of Zn in shoots (Figure 5a,b, Supplementary Materials 1 and 2). A negative correlation between the pH and solution Zn verified in the study was aligned with results reported in other work relating Zn supplying to coffee plants treated with variable rates of HA [10]. Another factor regulating Zn availability in soils is the OM and its pools; Zn interacts with organic radicals present in the OM structure, forming stable complexes capable of reducing Zn availability in soil [2]. 

However, the OM is also a source of soluble organic compounds capable of binding micronutrients through the formation of soluble organo-metal complexes [36]. In this study, RO, which had a higher OM content than YO, presented a lower soil Zn-DTPA content (maize experiment). However, RO had a greater Zn-DTPA content (Figure 1c) and produced a higher maize biomass and Zn in the shoot than the plants grown in YO (Figure 2). Thus, soil type is a key issue influencing the magnitude of plant response to Zn fertilization [2,4], as well as the role played by HA on plant nutrition and growth [8].

Regarding the soil’s physical and physicochemical properties, the texture (clay content), OM, and pH were the factors that sharply influenced soil Zn availability. Soil properties are capable of modulating Zn interaction, adsorption, and precipitation with soil components, including organic matter pools [1,2,4]. When associated with organic ligands, the Zn availability to crops relies on the chemical stability, solubility, and reactivity of the bonds formed between the humic matrix and the metal in the complexes formed [7,8]. Thus, RO, the soil with the highest OM and clay contents and the lowest pH is initially more prone to adsorb Zn than YO (Figure 1). 

However, soluble Zn and maize biomass production were higher in RO over YO, signaling the possible formation of soluble to complexes with natural OM fractions present in RO, a key issue to be considered to supply Zn to plants in cultivation conditions where HA and Zn are simultaneously added to the soil (Figure 1). A low supply of soluble Zn affects plant growth, mirrored by the increased root: shoot ratio of maize plants grown in the soil with low Zn contents in solution (Figure 1, Figure 2 and Figure 6a). Humic acid acts to relieve the stress of low contents of readily available Zn in YO, and reduces the maize root: shoot ratio, which is a signal of suitable conditions for plentiful plant growth.

An increase of the root: shoot ratio is strongly related to the capacity of the growth medium (soil) to nourish plants. In low-fertility soils, plants promote a greater release of C-exudates for root elongation, thus consuming energy that could be eventually driven to increase shoot biomass [37,38]. Increased root proliferation at the expense of shoot could result in a decrease in the plant above biomass. It is reported that an increase in the root: shoot ratio resembles the low levels of soluble Zn in the growth medium [38]. In this study, the negative correlation between soluble Zn and the root: shoot ratio was confirmed in the maize experiment (Figure 5, Supplementary Material 1), though the main factor of stress to plants was the increase of solution pH, followed by an increased root: shoot ratio, and reduced maize and brachiaria biomass production (Figure 5, Supplementary Materials 1 and 2).

Contrary to the maize experiment, even with a higher maize biomass production and Zn accumulation in RO over YO, the conditions of YO soil solution were essential to improve brachiaria growth (brachiaria) (Figure 3 and Figure 4). However, the soil Zn-DTPA was higher in RO over YO (Figure 3), indicating that the organic-Zn complexes in RO, naturally richer in OM than YO, possibly reduced the soil Zn-DTPA in the maize experiment; thus, less residual Zn was thought to be supplied to brachiaria plants. The effect aforementioned signals that the initial formation of Zn complexes with natural OM pools of RO is less stable over time, thus not suitable to meet Zn plant demand in the long term.

In the brachiaria experiment, the soil type effect on plants relies on HA application (Figure 3 and Figure 4). Despite the higher soil and solution pH of RO over YO, when brachiaria was cultivated in sequence to maize, the HA application reduced the solution pH in YO, with values similar to RO (Figure 3). This effect possibly eliminated the formation of insoluble forms of Zn as the soil pH was increased [4]. 

In addition to the soil type effect, HA increased the soluble Zn contents in the two Oxisols in both maize and brachiaria experiments, besides increasing soil Zn-DTPA contents (Figure 1 and Figure 3). The stoichiometry of the reaction between HA and Zn is an important factor regulating the amount of soluble Zn in Zn–HA complexes [7]. When Zn was added to soil at low concentration (5 mg kg^−1^), mainly in soils with low OM contents, the use of 50 mg kg^−1^ C as HA reduced Zn in soil solution, which was mainly due to the specific adsorption and formation of Zn–HA complexes of high stability, reducing Zn uptake by crops [9]. However, in this study, with an adequate concentration of Zn (20 mg kg^−1^) added to soil, it was demonstrated that HA acts positively on Zn contents in soil solution, as well as on crop Zn nutrition (Figure 1 and Figure 3).

In solution, the main forms of Zn found are free hydrated Zn(6H_2_O)^2+^ and soluble organic complexes. When HA is applied together with Zn sulfate, Zn free ions become partially complexed by HA radicals, as observed by the FTIR spectra, which demonstrated the production of aqueous Zn-complexes and the formation of RSO_4_^−^M^+^ bonds (Figure 6). The aforementioned factors contribute to an increase in the Zn content in the soil solutions of both Oxisols due to Zn’s bond to organic molecules such as HAs, thus contributing to the improvement of Zn availability in soil [4,8,15]. When Zn interacts with organic molecules, there are new forms of Zn capable of generating in soil soluble Zn chemical species, which improve Zn diffusion from solution to plant roots [4]. The increased release of Zn from the solid phase to soil solution in response to HA application was already demonstrated elsewhere [9]. Although the Zn in the soil solution is a small fraction of soil total Zn, this is the Zn pool readily available to plants; thus, Zn in soil solution is a key issue regulating Zn uptake by crops [1,4,26].

The role played by HA on soil physicochemical properties and nutrient available contents are claimed as indirect effects of HS on plants. Humic acid changes the growing medium in a way that plant growth is positively affected [8,9,11,15,16]. Besides the indirect effect, in contact with plant tissues, HA plays a positive role in crop nutrition and growth [11,12]. The direct effects of HS on plants include increased H^+^-ATPase membrane activity, improved cell plasmatic membrane permeability, regulation of the levels and activity of reactive oxygen species (ROS), increased activity of nutrient transporters, control of nutrient translocation, and regulation of several plant physiological processes [8,9,11,12,13,15,16].

Recently, it was demonstrated that metal-HA complexes are a source of readily available nutrients, besides increasing the bioactivity of humic molecules on plants [20]. Thus, the direct and indirect effects of HA occur simultaneously, improving plant nutrition, growth, and yield [9,11,15,20]. In this study, the potential direct effects of HA on plants are indicated by the presence of peaks at 2915, 2845, 1560, and 1365 cm^−1^ in the FTIR spectra when HA was mixed with ZnSO_4_ (Figure 4). These FTIR spectral bands are indices of HA bioactivity on plants, considering that these FTIR spectral fingerprints were positively related to improved lateral root emergence in maize plants [14].

When HA was combined with Zn fertilization in RO, the maize biomass production was improved and positively related to Zn contents in soil solution. However, Zn in maize shoot was not augmented (Figure 1 and Figure 2). Despite the indirect effect of HA on soil solution attributes, both direct and indirect (soil) effects of HA on plants occurred simultaneously, considering that the increased availability of Zn in soil solution did no improve plant Zn uptake, even with the increase in maize biomass production. HA positively affected brachiaria growth in RO, improving root proliferation and Zn uptake (Figure 3 and Figure 4). When HA was added to YO, the brachiaria biomass increased, and plant Zn nutrition was improved (Figure 4). This effect of HA on plants is more pronounced in YO over RO (Figure 4).

The results reported in this study showed that HA promoted positives effects on maize, and guaranteed a residual Zn for brachiaria plants grown in sequence to maize, in a soil type-dependent way. However, maize–brachiaria cultivated in a sequence is widely performed in Brazilian cropping areas [3]; there is no study reporting how HA-Zn interaction exerts influence on the brachiaria cultivated in sequence to maize. Evaluating only the effect of HA-Zn interaction in single cultivation of coffee seedlings, Just et al. [10] reported that the application of 50 mg kg^−1^ C-HA increased the solution Zn in alkalinized soils (pH > 7.2), probably due to the Zn–HA complexation. In this study, the main and residual effects of Zn fertilization+HA are related to the increase in the solution Zn contents. In addition, in the soil with the lower OM content (YO), HA acted as a buffer for soil acidification, thus, favoring Zn acquisition and growth of brachiaria cultivated in sequence to maize.

The results of this study showed that humic increased the release of Zn from Oxisols, mainly to soil solution, increased Zn availability, transport and uptake by crops. Release of Zn relies on HA concentration, solution pH, and soil types, all of which influenced the degree of Zn complexation, precipitation, and amount of Zn moving from solid to liquid soil phase [39]. Overall, precipitation reactions, formation of Zn–HA complexes, and adsorption of humic acid to soil components reduce the amount of Zn released from soil [39]. Parent materials of highly weathered Brazilian soils are poor natural sources of Zn. Thus, both under natural vegetation and cultivation conditions, the Brazilian tropical soils are limited in their capacity to nourish grasses (high-demanding Zn crops) with adequate Zn levels. Even in enriched OM soils, organic compounds are chemically stabilized or bonded to clay and Fe and Al oxides in tropical soils. Thus, it is suitable to add some exogenous humic materials to improve Zn nutritional status and growth of maize-brachiara in Brazilian crop fields. Successive cultivation of maize–brachiaria is very common in the large area of crop-livestock integrated systems established in Brazil, and brachiaria usually is supposed to cope with the residual Zn derived from the fertilization of the first crop of rotation. In this study, it was demonstrated that the Zn soil chemistry and forms are affected by the presence of Zn–HA complexes in highly weathered soils, with a positive effect on Zn uptake and growth of maize and brachiaria. In line with Boguta et al. (6), in a soil type-dependent way, the understanding of Zn–HA interactions is the first step to improve Zn fertilization in tropical soils and a suitable strategy to be used in the synthesis of Zn–HA fertilizers.

## 5. Conclusions

FTIR spectroscopy revealed the complexation of Zn sulfate by HA through sulfone and carbon functional groups. A significant portion of Zn fertilizer applied to Oxisols is not available for the first crop (maize), but it is prone to be acquired by brachiaria plants. It was demonstrated that Zn in solution is sharply increased by the combined use of Zn sulfate and HA. The humic acid that was complexed to ZnSO_4_ increased Zn contents in Oxisols solution while reducing the negative reactions (e.g., adsorption and precipitation) of Zn in the whole soil. In the lower organic matter Oxisol, the use of HA is capable of assuring adequate supplying of residual Zn and increasing growth of brachiaria grown in sequence to maize. Oxisol organic matter content is a key factor regulating the action of HA on soil Zn availability and plant growth.

## Figures and Tables

**Table 1:** Main chemical and physicochemical properties of Oxisols in which maize and brachiaria were cultivated.

Oxisol	pH	C (g kg^−1^)	Clay (g kg^−1^)	Silt (g kg^−1^)	Sand (g kg^−1^)	Available Zn (DTPA) (mg kg^−1^)
Red (RO)	6.4	19.8	770	100	130	0.93
Yellow (YO)	6.2	4.49	460	85	455	0.60

pH was determined in water at a ratio of 1:2.5 (*w*/*v*); C–total C determined (dry combustion) in an automatic TOC analyzer., silt and sand were determined by the Boyoucos method; Zn was determined by ICP-OES after extraction of Zn from the soil through the use of the DTPA extraction method.

## Data Availability

Not applicable.

## Supplementary Materials

The following are available online. Supplementary Material 1: The Pearson’s linear correlation matrix for soil attributes and maize traits. * and ** significant correlation of soil and plant attributes at *p* < 0.05 and *p* < 0.01, respectively; blue ellipse with right sloping top: positive correlation; red ellipse with left sloping top: negative correlation. SDM, RDM and TDM: shoot, root and total dry matter production, respectively; R:S ratio: ratio of shoot (SDM) over root dry matter (RDM); Zn accumulation: Zn accumulated in maize shoot. Supplementary Material 2 The Pearson’s linear correlation matrix for soil attributes and brachiaria traits. * and ** significant relationships between soil and plant traits at *p* < 0.05 and *p* < 0.01, respectively; blue ellipse with right sloping top: positive correlation; red ellipse with left sloping top: negative correlation. SDM, RDM and TDM: brachiaria shoot, root and total dry matter production, respectively; R:S ratio: ratio of shoot (SDM) over root dry matter (RDM); Zn accumulation: Zn accumulated in brachiaria shoot.

## Sample Availability

Samples of humic acid, ZnSO_4_·7H_2_O and the mixture of humic acid with ZnSO_4_·7H_2_O are available from the authors.

## References

[1] Broadley M., Brown P., Cakmak I., Rengel Z., Zhao F., Marschner H. (2012). Function of nutrients: Micronutrients. Mineral Nutrition of Higher Plants.

[2] Alloway B.J. (2009). Soil factors associated with zinc deficiency in crops and humans. Environ. Geochem. Health.

[3] Anghinoni G., Tormena C.A., Lal R., Zancanaro L., Kappes C. (2019). Enhancing soil physical quality and cotton yields through diversification of agricultural practices in central Brazil. Land Degrad. Dev..

[4] Montalvo D., Degryse F., da Silva R.C., Baird R., McLaughlin M. (2016). Agronomic Effectiveness of Zinc Sources as Micronutrient Fertilizer.

[5] Lopes A., Guilherme L.R.G. (2016). A Career Perspective on Soil Management in the Cerrado Region of Brazil. Adv. Agron..

[6] Stevenson F.J. (1994). Humus Chemistry: Genesis, Composition, Reactions.

[7] Boguta P., Sokołowska Z. (2016). Interactions of Zn(II) Ions with Humic Acids Isolated from Various Type of Soils. Effect of pH, Zn Concentrations and Humic Acids Chemical Properties. PLoS ONE.

[8] Rose M.T., Patti A.F., Little K.R., Brown A.L., Jackson W.R., Cavagnaro T.R. (2014). A Meta-Analysis and Review of Plant-Growth Response to Humic Substances. Adv. Agron..

[9] de Morais E.G., Silva C.A., Maluf H.J.G.M. (2021). Soaking of Seedlings Roots in Humic Acid as an Effective Practice to Improve Eucalyptus Nutrition and Growth. Commun. Soil Sci. Plant Anal..

[10] Justi M., Morais E., Silva C.A. (2019). Fulvic acid in foliar spray is more effective than humic acid via soil in improving coffee seedlings growth. Arch. Agron. Soil Sci..

[11] Olaetxea M., de Hita D., Garcia C.A., Fuentes M., Baigorri R., Mora V., Garnica M., Urrutia O., Erro J., Zamarreño A.M. (2018). Hypothetical framework integrating the main mechanisms involved in the promoting action of rhizospheric humic substances on plant root- and shoot- growth. Appl. Soil Ecol..

[12] Tavares O., Santos L., Ferreira L., Sperandio M., Da Rocha J., García A.C., Dobbss L., Berbara R., De Souza S., Fernandes M. (2016). Humic acid differentially improves nitrate kinetics under low- and high-affinity systems and alters the expression of plasma membrane H+-ATPases and nitrate transporters in rice. Ann. Appl. Biol..

[13] Jindo K., Martim S.A., Navarro E.C., Pérez-Alfocea F., Hernandez T., Garcia C., Aguiar N.O., Canellas L.P. (2012). Root growth promotion by humic acids from composted and non-composted urban organic wastes. Plant Soil.

[14] Aguiar N., Novotny E., Oliveira A., Rumjanek V., Olivares F.L., Canellas L. (2013). Prediction of humic acids bioactivity using spectroscopy and multivariate analysis. J. Geochem. Explor..

[15] Nardi S., Schiavon M., Francioso O. (2021). Chemical Structure and Biological Activity of Humic Substances Define Their Role as Plant Growth Promoters. Molecules.

[16] Shah Z.H., Rehman H.M., Akhtar T., Alsamadany H., Hamooh B.T., Mujtaba T., Daur I., Al Zahrani Y., Alzahrani H.A.S., Ali S. (2018). Humic Substances: Determining Potential Molecular Regulatory Processes in Plants. Front. Plant Sci..

[17] De Morais E.G., Silva C.A., Rosa S.D. (2018). Nutrient acquisition and eucalyptus growth affected by humic acid sources and concentrations. Semin. Ciênc. Agrár..

[18] Rosa S.D., Silva C.A., Maluf H.J.G.M. (2018). Wheat nutrition and growth as affected by humic acid-phosphate interaction. J. Plant Nutr. Soil Sci..

[19] Pinheiro P.L., Passos R.R., Peçanha A.L., Canellas L.P., Olivares F.L., Mendonça E.D.S. (2018). Promoting the growth of Brachiaria decumbens by humic acids (HAs). Aust. J. Crop. Sci..

[20] Zanin L., Tomasi N., Cesco S., Varanini Z., Pinton R. (2019). Humic Substances Contribute to Plant Iron Nutrition Acting as Chelators and Biostimulants. Front. Plant Sci..

[21] Socrates G. (2004). Infrared and Raman Characteristic Group Frequencies: Tables and Charts.

[22] Sadeek S.A. (1993). Preparation, infrared spectrum and thermal studies of [Zn2(H2O)4(SO4)2] complex formed by the reaction of urea with zinc (II) sulphate. J. Phys. Chem. Solids.

[23] Saha J., Podder J. (2011). Crystallization Of Zinc Sulphate Single Crystals And Its Structural, Thermal And Optical Characterization. J. Bangladesh Acad. Sci..

[24] Swift R.S. (1996). Organic Matter Characterization. Methods of Soil Analysis: Part 3 Chemical Methods.

[25] Álvarez J.M. (2007). Influence of Soil Type on the Mobility and Bioavailability of Chelated Zinc. J. Agric. Food Chem..

[26] do Carmo D.L., Silva C.A., De Lima J.M., Pinheiro G.L. (2016). Electrical Conductivity and Chemical Composition of Soil Solution: Comparison of Solution Samplers in Tropical Soils. Rev. Bras. Cienc. Solo.

[27] Brown A.L., Quick J., Eddings J.L. (1971). A Comparison of Analytical Methods for Soil Zinc. Soil Sci. Soc. Am. J..

[28] Kalra Y. (1997). Handbook of Reference Methods for Plant Analysis.

[29] Gautam R., Vanga S., Ariese F., Umapathy S. (2015). Review of multidimensional data processing approaches for Raman and infrared spectroscopy. EPJ Tech. Instrum..

[30] Mendiburu F. (2010). Agricolae: Statistical Procedures for Agricultural Research. https://www.researchgate.net/publication/303256192_Agricolae_Statistical_Procedures_for_Agricultural_Research.

[31] R Core Team (2020). R: A Language and Environment for Statistical Computing.

[32] Wickham H., Averick M., Bryan J., Chang W., McGowan L.D., François R., Grolemund G., Hayes A., Henry L., Hester J. (2019). Welcome to the Tidyverse. J. Open Source Softw..

[33] Wei T., Simko V. (2017). R Package “Corrplot”: Visualization of a Correlation Matrix. https://cran.r-project.org/web/packages/corrplot/corrplot.pdf.

[34] Kassambara A., Mundt F. (2020). Factoextra: Extract and Visualize the Results of Multivariate Data Analyses. https://rdrr.io/cran/factoextra/.

[35] Lê S., Josse J., Husson F. (2008). FactoMineR: AnRPackage for Multivariate Analysis. J. Stat. Softw..

[36] Wang K., Xing B. (2005). Structural and Sorption Characteristics of Adsorbed Humic Acid on Clay Minerals. J. Environ. Qual..

[37] Ikka T., Ogawa T., Li D., Hiradate S., Morita A. (2013). Effect of aluminum on metabolism of organic acids and chemical forms of aluminum in root tips of Eucalyptus camaldulensis Dehnh. Phytochemistry.

[38] Morais E., Silva C.A., Maluf H.J.G.M. (2020). UV-visible Spectroscopy as a New Tool to Predict the Bioactivity of Humic Fragments Induced by Citric/ Oxalic Acids on Eucalyptus Nutrition and Growth. Commun. Soil Sci. Plant Anal..

[39] Özkaraova Güngör E.B., Bekbolet M. (2010). Zinc release by humic and fulvic acid as influenced by pH, complexation and DOC sorption. Geoderma.

