# Supplementary material for: Humic Acid Improves Zn Fertilization in Oxisols Successively Cultivated with Maize–Brachiaria
Source: Molecules. 2021 Jul 29;26(15):4588. doi: 10.3390/molecules26154588 (PMC8348479; doi:10.3390/molecules26154588)
Supplement: Supplementary file 1 [file molecules-26-04588-s001.zip › molecules-1296603-supplementary.pdf]

Supplementary material

Supplementary material 1. The Pearson's linear correlation matrix for soil attributes and maize traits. * and ** significant relationship of soil and plant attributes at $p < 0.05$ and $p < 0.01$, respectively; blue ellipse with right sloping top: positive relationship; red ellipse with left sloping top: negative correlation. SDM, RDM and TDM: shoot, root and total dry matter production, respectively; R:S ratio: ratio of shoot over (SDM) over root dry matter (RDM); Zn accumulation: Zn accumulated in maize shoots.

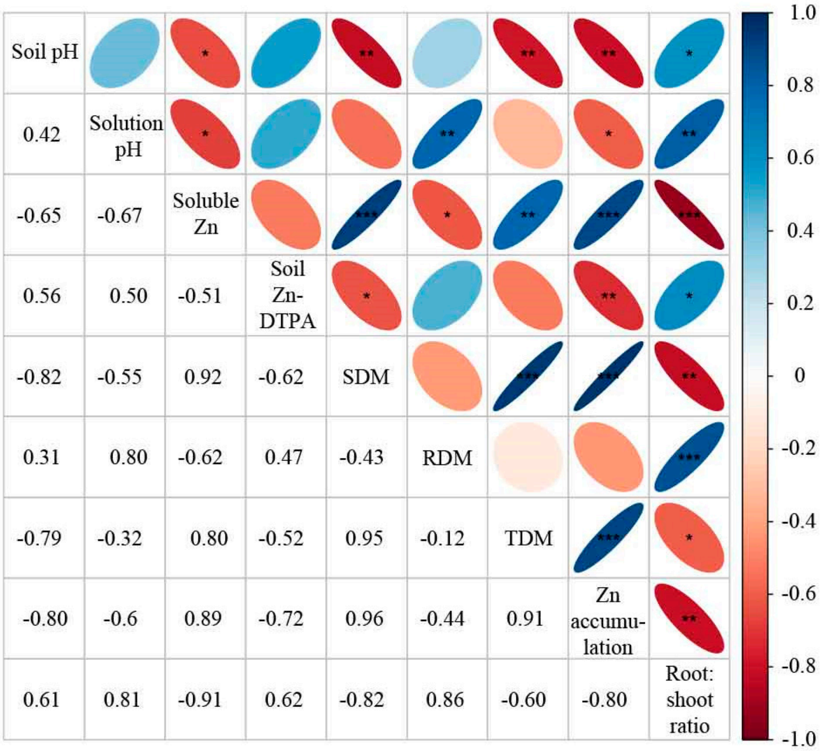

Supplementary material 2. The Pearson's linear correlation matrix for soil attributes and brachiaria traits. * and ** significant relationhips between soil and plant traits at $p < 0.05$ and $p < 0.01$, respectively; blue ellipse with right sloping top: positive correlationship; red ellipse with left sloping top: negative correlationship. SDM, RDM and TDM: brachiaria shoot, root and total dry matter production, respectively; R:S ratio: ratio of shoot (SDM) over root dry matter (RDM); Zn accumulation: Zn accumulated in brachiaria shoots.

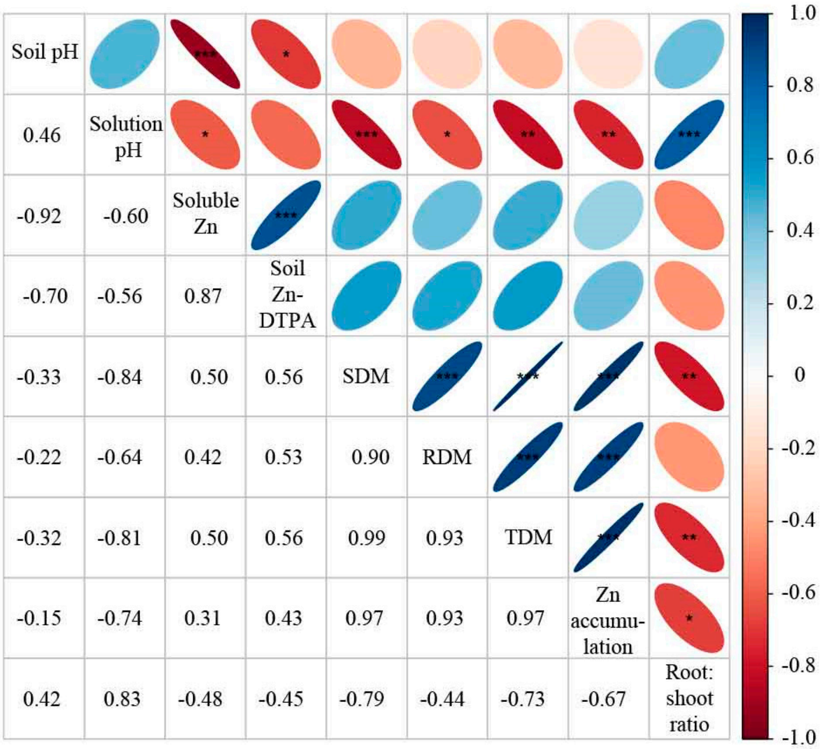
Soil pH
0.46 Solution pH
-0.92 -0.60 Soluble Zn
-0.70 -0.56 0.87 Soil Zn-DTPA
-0.33 -0.84 0.50 0.56 SDM
-0.22 -0.64 0.42 0.53 0.90 RDM
-0.32 -0.81 0.50 0.56 0.99 0.93 TDM
-0.15 -0.74 0.31 0.43 0.97 0.93 0.97 Zn accumu-lation
0.42 0.83 -0.48 -0.45 -0.79 -0.44 -0.73 -0.67 Root: shoot ratio
1.0
0.8
0.6
0.4
0.2
0
-0.2
-0.4
-0.6
-0.8
-1.0
